# A Personal Note on “Retraction Notes”: Distinguishing Author-Offered Retractions from Other Causes

**DOI:** 10.31662/jmaj.2025-0364

**Published:** 2025-09-12

**Authors:** Shigeki Matsubara

**Affiliations:** 1 Department of Obstetrics and Gynecology, Jichi Medical University, Tochigi, Japan; 2 Department of Obstetrics and Gynecology, Koga Red Cross Hospital, Koga, Ibaraki, Japan; 3 Medical Examination Center, Ibaraki Western Medical Center, Chikusei, Ibaraki, Japan

**Keywords:** honest error, manuscript, misconduct, paper, retraction

## To the Editor,

A recent announcement from the Editor-in-Chief (EIC) of the *JMA Journal* ^(1)^ has reminded us: retraction notes are crucial for the transparency of the academic world. I raise two issues: when the author voluntarily requests retraction, the note’s title should indicate so; and its content should be readable on PubMed. These would allow readers to better understand the retraction’s context.

Although there may be no “good retraction,” voluntary retraction differs from retraction due to misconduct. As a seasoned obstetrician, I can appropriately interpret retraction notes in obstetrics, which may have no special issues in this context. I searched PubMed for “retraction and obstetrics,” yielding 1,622 papers (May 21, 2025). I reviewed the most recent 100, of which 60 were “Retraction Note.” Reasons included data mishandling (n = 12), plagiarism (9), image mishandling (9), methodological flaws (9), duplicate publication (6), inappropriate institutional review board approval (6), and others (3), totaling 54. The remaining six were by “author’s request”: in three, following concerns raised by others; in the other three, authors independently requested retraction. These three (5%: 3/60) suggest that the authors recognized their mistakes and made them public, rather than hoping time would erase the paper. While their “unpreparedness” may be criticized, I wish to recognize that “to err is human.”

This type of retraction should be noted in the title―e.g., “Retraction Note [Authors’ Request].” Such a title makes readers grasp the context. This may foster an atmosphere where, once mistakes are acknowledged, authors feel encouraged to consult the journal about a voluntary retraction. The ICMJE guideline ^(2)^ states: “retraction with republication can be considered” when honest error leads to major changes in the conclusion. Even then, prompt reporting is essential, as “these corrections may have less impact on science” once the data becomes outdated ^(2)^.

PubMed should display the content of retraction notes, just as it does abstracts. While some are already readable, others are not. When a journal is not open access and PubMed omits the note, readers cannot know the reason. Understanding the reasons helps, especially for younger readers learning publication ethics―a point I believe the *JMA Journal*’s EIC also intended ^(1)^. It may also save authors who have voluntarily retracted. Of the six voluntary retractions, one was from a non-open access journal, and PubMed did not show its content.

These proposals may help younger readers learn publication ethics ^(3)^, reduce fruitless “you did/I did not” debates, and promote transparency in academic writing―an increasingly important value in the artificial intelligence era^(3), (4)^.

## Article Information

### Acknowledgments

I thank doctors Daisuke Matsubara (Division of Community and Family Medicine and Department of Pediatrics, Jichi Medical University, Tochigi, Japan) and Yuri Matsubara (Department of Public Health, Dokkyo Medical University, Tochigi, Japan) for their help.

### Author Contribution

Shigeki Matsubara designed this study and wrote, edited, and approved the final manuscript. Shigeki Matsubara meets the ICMJE criteria for authorship.

### Conflicts of Interest

None

### Ethics Approval

Jichi Medical University does not demand IRB approval for this type of study. Patient anonymity preservation and informed consent for reporting are not applicable.

### Data Availability Statement

Data sharing is not applicable to this article as no new data were created or analyzed in this study.

## References

[1] Announcement from the Editor-in-Chief - translations may constitute duplicate publication [Internet]. JMA Journal. 2025 [cited 2025 Jul 22]. Available from: https://www.jmaj.jp/static.php?d=250627&c=news

[2] ICMJE recommendations for the conduct, reporting, editing, and publication of scholarly work in medical journals. Corrections and version control [Internet]. ICMJE (International Committee of Medical Journal Editors). 2025 [cited 2025 Jul 22]. Available from: https://www.icmje.org/recommendations/browse/publishing-and-editorial-issues/corrections-and-version-control.html

[3] Matsubara S. Post-publication concerns and artificial intelligence. BJOG. 2025. Apr 8. doi: 10.1111/1471-0528.18165.10.1111/1471-0528.1816540196927

[4] Matsubara S, Matsubara D. Research misconduct: use of generative artificial intelligence in writing may lower the threshold. Eur J Obstet Gynecol Reprod Biol. 2025;304:172-3. 39609114 10.1016/j.ejogrb.2024.11.038

